# The Mitochondrial Genome of *Amara aulica* (Coleoptera, Carabidae, Harpalinae) and Insights into the Phylogeny of Ground Beetles

**DOI:** 10.3390/genes11020181

**Published:** 2020-02-09

**Authors:** Zhenya Li, Xinxin Li, Nan Song, Huiji Tang, Xinming Yin

**Affiliations:** 1 College of Plant Protection, Henan Agricultural University, Zhengzhou 450002, China; zhenya0371@163.com (Z.L.); lixinxin412@126.com (X.L.); xinmingyin@hotmail.com (X.Y.); 2 Technical Center, Zhengzhou Customs District, Zhengzhou 450002, China; huijit@tom.com

**Keywords:** ground beetle, mitogenome, next-generation sequencing, phylogeny

## Abstract

Carabidae are one of the most species-rich families of beetles, comprising more than 40,000 described species worldwide. Forty-three complete or partial mitochondrial genomes (mitogenomes) from this family have been published in GenBank to date. In this study, we sequenced a nearly complete mitogenome of *Amara aulica* (Carabidae), using a next-generation sequencing method. This mitogenome was 16,646 bp in length, which encoded the typical 13 mitochondrial protein-coding genes, 22 transfer RNA genes, two ribosomal RNA genes, and a putative control region. Combining with the published mitogenomes of Carabidae and five outgroup species from Trachypachidae, Gyrinidae and Dytiscidae, we performed phylogenetic estimates under maximum likelihood and Bayesian inference criteria to investigate the phylogenetic relationships of carabid beetles. The results showed that the family Carabidae was a non-monophyletic assemblage. The subfamilies Cicindelinae, Elaphrinae, Carabinae, Trechinae and Harpalinae were recovered as monophyletic groups. Moreover, the clade (Trechinae + (Brachininae + Harpalinae)) was consistently recovered in all analyses.

## 1. Introduction

The Carabidae, also known as carabid beetles or ground beetles, are among the most species-rich families in Caraboidea. They currently comprise more than 40,000 described species worldwide, which can be classified into 16 subfamilies [1] and 86 tribes [2,3,4]. Carabid beetles are often considered as indicators of ecological changes, and are used as the biocontrol agents against insect pests in crops [5,6,7]. Furthermore, some researches indicated that carabids could contribute to weed management in agroecosystems (as reviewed in [8]). 

The taxonomy of carabid beetles has been extensively studied. Traditionally, phylogenetic reconstructions of carabids are based on the morphological characters, for example, the male [9,10] and female genitalia [11] and the wing folding structures [12]. Liebherr and Will (1998) recovered Carabidae as a non-monophyletic assemblage, with the characters of the female reproductive tract [13]. By analyzing the larval morphology, Arndt (1998) retrieved Carabidae as a monophyletic group, with the members of Rhysodidae excluded [14]. Kavanaugh (1998) investigated the relationships among the basal carabids and recovered Trachypachidae as sister to all carabid taxa [15]. The Cicindelinae (tiger beetles) was found to be related to the tribes Carabini, Cychrini, Cicindelini and Omophronini [15]. Grebennikov and Maddison (2005) analyzed the phylogenetic relationships within the supertribe Trechitae based on larval morphology [16]. Beutel et al. (2006) applied morphological characters of adults and larvae to recover Carabidae as a sister to *Omoglymmius* (Rhysodidae), which together form a sister group of Trachypachidae [17]. Studies on morphology of defense glands [18,19,20] and those on karyotypes [21,22] of some carabid species also contributed to understanding of the phylogeny of Carabidae.

Molecular data can be used to address problems when morphological evidence have been conflicting or difficult to interpret. Based on *18S rDNA* sequences, Maddison et al. (1999) supported Carabidae (including cicindelines, rhysodines and paussines) as monophyletic and that Brachinini probably was a sister group to Harpalinae [23]. Their results also assumed Harpalinae, Cicindelinae, Rhysodinae and Paussinae to be closely related to each other. However, their further analyses based on expanding molecular data (*18S rDNA*, *28S rDNA* and *wingless* gene) recovered Carabidae as non-monophyletic, with respect to the trachypachid beetles [24]. 

Gough et al. (2019) recovered Cicindelinae as a sister group to the subfamily Rhysodinae, and placed the tribe Megacephalini nested within Platychilini in Cicidelinae [25]. Maddison et al. (2019) inferred the phylogeny of the supertribe Trechitae based on two nuclear ribosomal genes (*18S rDNA* and *28S rDNA*) and four nuclear protein-coding genes (*wingless* gene, carbamoyl phosphate synthetase domain of the rudimentary gene, arginine kinase gene and muscle-specific protein 300 gene) [26]. In addition, some molecular studies had attempted the phylogenetic reconstructions at the genus or subgenus levels (*Bembidion*: Maddison, 2012; *Carabus*: Deuve et al., 2012; *Ohomopterus*: Sota and Vogler, 2003; *Pamborus*: Sota et al., 2005; *Paraphaenops*: Ortuño et al., 2017; *Pterostichus*: Sasakawa and Kubota, 2007) [27,28,29,30,31,32].

Recent studies on the suborders of Coleoptera or on the whole Coleoptera phylogeny also involved the exemplars of Carabidae. Hunt et al. (2007) [33] suggested that the monophyletic Geadephaga (comprising Trachypachidae, Rhysodidae and Carabidae including cicindelines) [34] formed a sister group to (Hydradephaga + Derodontoidea). At the subfamily level, the Harpalinae was strongly supported as a sister group to Paussinae, while the Cicindelinae was placed in a derived position and sister to a clade of (Rhysodinae + Migadopinae) [33]. Bocak et al. (2014) recovered the monophyletic Cicindelinae as a sister group to Haliplidae [35]. Timmermans et al. (2016) supported Carabidae as non-monophyletic and that tiger beetles were recognized as a separate family (namely Cicindelidae) [36]. Crampton-Platt et al. (2015) clustered the families Carabidae, Tenebrionidae, Coccinellidae and Ptilodactylidae in a clade to form the superfamily Caraboidea, which is sister to Archostemata [37]. In the study of Mckenna et al. (2015), the monophyletic Geadephaga was retrieved as sister to Hydradephaga, whereas the Carabidae was shown to be non-monophyletic with respect to Trachypachidae and Rhysodidae [38]. Baca et al. (2017) inferred Hydradephaga as a paraphyletic group, with Gyrinidae sister to Geadephaga (containing families Carabidae and Trachypachidae) [39]. López-López and Volger (2017) supported Geadephaga and Hydradephaga as two independent lineages based on the mitogenomic data, and recovered cicindelids and trachypachids as sister to all other Geadephaga [40]. Moreover, the authors suggested that the groups of cicindelids and trachypachids deserved the family status, namely, the Cicindelidae and Trachypachidae. Zhang et al. (2018) supported the monophyly of Carabidae and the most-basal position of *Cicindela* (Cicindelinae) within Carabidae [41]. In summary, resolving the phylogenetic relationships among these taxonomic groups is important and deserves further investigation. 

The harpaline carabid beetles (Carabidae, Harpalinae) diversified rapidly during the Cretaceous period [42,43]. The Harpalinae includes more than 19,000 described species in the world [44], which is the largest subfamily of Carabidae. Harpalines are in appearance, anatomy, ecology and behavior a highly diverse group. The monophyly of Harpalinae seems uncontentious. Morphological characters uniting harpalines have been summarized in the study of Ober (2002) [45]. Some molecular studies recovered Harpalinae as a monophyletic group [4,23,45]. However, in the analysis of [24], Harpalinae was retrieved as non-monophyletic due to the embedded placement of Brachinini (Carabidae: Brachininae). In addition, the tribe Lebiini in Harpalinae was proposed as the rank of subfamily (Lebiinae) by some authors [46,47]. 

In recent years, sequences of mitochondrial genome (mitogenome) have been widely used to investigate insect phylogenetic relationships, molecular evolution and conservation genetics [36,37,48,49,50,51,52]. As a class of molecular marker, the mitogenome has the characteristics of maternal inheritance, rapid evolution rate, simple genetic structure and rare recombination [53]. The typical insect mitochondrial genome is a closed-circular and double-stranded DNA molecule of nearly 16 kb in length, and contains 13 protein-coding genes (PCGs), 22 transfer RNA (tRNA) genes, two ribosomal RNA (rRNA) genes and one large AT-rich noncoding control region. The mitogenome provides an increasingly complete picture of phylogenetic relationships of insects through a large number of taxon sampling [51,54]. With the development of sequencing technology, next-generation sequencing (NGS) provides a much more cost-effective and time-saving method to generate a great number of mitogenome sequences simultaneously [37,51,55].

In this paper, we sequenced the nearly complete mitogenome of *Amara aulica* from the subfamily Harpalinae, by using a next-generation sequencing method. Combined with other 48 published mitogenome sequences, we reconstructed the phylogenetic relationships of the main lineages in Carabidae, under the maximum likelihood (ML) and Bayesian inference (BI) criteria.

## 2. Materials and Methods

### 2.1. Sampling and DNA Extraction

The species *A. aulica* is native to Europe and has been introduced to Asia and North America [56,57,58]. Adult specimens were collected from Zhengzhou, Henan Province (the geospatial coordinates: 34.723° N, 113.635° E). No specific permits were required for the insects sampled for this study. 

After the samples were directly killed and preserved in absolute ethanol, they were stored in the dark at −20 °C in Entomological Museum of Henan Agricultural University (voucher number: EMHAU-2015-Zz122902) for further experiment. Total genomic DNA of the individual specimen was extracted from the thorax with the TIANamp Micro DNA kit (TIANGEN BIOTECH CO., LTD, Beijing, China), following the manufacturer’s instructions.

### 2.2. Mitochondrial Genome Sequencing and Assembly

Next-generation-sequencing (NGS) technology was applied to obtain the mitogenome sequences. Genomic DNA was pooled with other insect species, which had a distantly phylogenetic relationship to *A. aulica*. In the pool, the DNA concentrations were approximately equimolar. The library was constructed by using the Illumina TruSeqTM DNA Sample Prep Kit (Illumina, San Diego, CA, USA), with the insert size of 350 bp. Following sequencing was conducted on an Illumina HiSeq2500 platform at Shanghai OE Biotech CO., LTD, with the 150-base paired-end strategy.

NGS QC toolkit [59] was used to filter raw data for quality control. The high-quality reads were assembled using IDBA-UD v. 1.1.1 (Hong Kong, China) [60], with the following settings: the minimum size of contig of 200, an initial k-mer size of 40, an iteration size of 10 and a maximum k-mer size of 90. Three mitochondrial gene fragments (*cox1*, *cob* and *rrnL*) were pre-sequenced for bait sequences, by using traditional polymerase chain reaction and Sanger sequencing. The primers for the polymerase chain reactions were used as those in Song et al. (2016b) [61]. The local-blasting searches were implemented in BioEdit [62], in order to identify the mitochondrial contig. 

### 2.3. Mitogenome Annotation and Analysis

**Figure 1:**
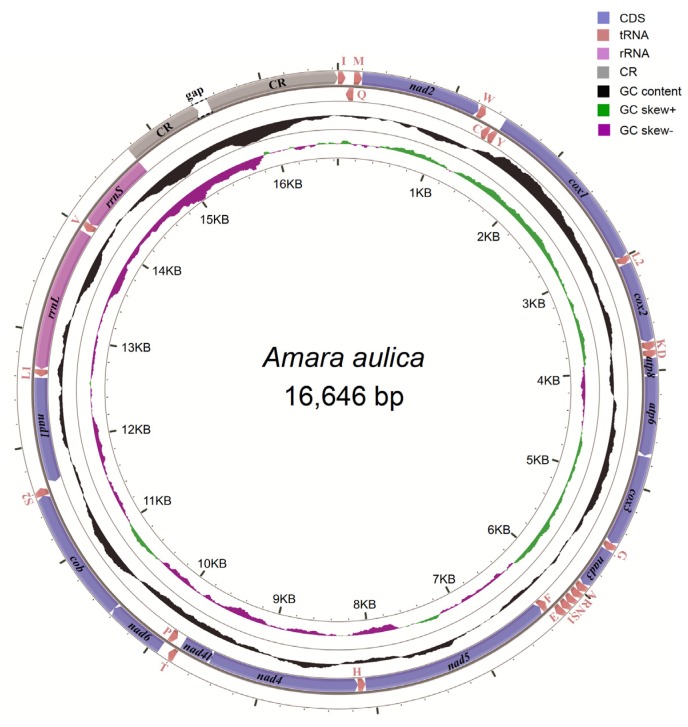
The structure of the mitochondrial genome of *Amara aulica*. Arrows indicate the direction of gene transcription. The inner circles show the GC content and the GC-skew values.

The initial mitogenome annotation was conducted in MITOS web [63]. The start codon, stop codon and length of each protein-coding gene were further checked and adjusted by alignment to the published carabid beetle mitogenomes in GenBank. The mitogenome organization of *A*. *aulica* was presented in Table 1. The genome structure image was generated in CGView (http://stothard.afns.ualberta.ca/cgview_server/) (Figure 1). The composition skew was calculated based on the AT-skew = (A − T)/(A + T) and GC-skew = (G − C)/(G + C) formulas [64]. The newly determined mitogenome sequence of *A. aulica* was deposited in GenBank, accession number MN335930. 

### 2.4. Sequence Alignment

Our taxon sample included 49 beetle mitogenome sequences representing 12 subfamilies of Carabidae (44 taxa) and three families of Trachypachidae, Gyrinidae and Dytiscidae as outgroups (five taxa) (Table 2). The protein-coding genes were aligned separately using TranslatorX [65] with the following parameters: Genetic code = “invertebrate mitochondrial”, Protein alignment = “MAFFT”, and the stop codons were excluded. Both the mitochondrial tRNA and rRNA genes were aligned using the program MAFFT under the iterative refinement method of “E-INS-i” [66]. The alignments were checked in MEGA 7 [67] and ambiguously aligned positions were manually excluded. Gaps were pruned using the online version of Gap Strip/Squeeze v2.1.0, with 40% Gap tolerance. Finally, the resulting alignments were concatenated together to make the dataset of PCGRNA (including 13 protein-coding genes, 22 tRNA genes two and rRNA genes), with the Perl script FASconCAT_v1.0 [68]. The mean *ka* (nonsynonymous substitution rate) and *ks* (synonymous substitution rate) values were calculated using DnaSP version 5 (Barcelona, Spain) [69].

### 2.5. Phylogenetic Analyses

In the phylogenetic analyses, our taxon sample included 46 beetle species representing 12 subfamilies of Carabidae, namely, Brachininae, Broscinae, Carabinae, Cicindelinae, Elaphrinae, Harpalinae, Nebriinae, Paussinae, Promecognathinae, Rhysodinae, Scaritinae and Trechinae. In addition, two mitogenome sequences from Dytiscidae and Gyrinidae respectively, and one from Trachypachidae were selected as outgroups. A total of 49 mitogenome sequences representing the taxa described above were compiled to make the data matrix of 49taxa_PCGRNA.

Phylogenetic trees were built based on the dataset of 49taxa_PCGRNA, under the maximum likelihood and Bayesian inferences. Maximum likelihood analysis was carried out using IQ-TREE [70] and applied the data partition schemes and best-fitting models pre-determined by PartitionFinder 2 [71] (Table S1). The data blocks were defined by genes and codon positions. Branch support was assessed using fast bootstrap analysis with 10,000 replicates. The Bayesian analysis was performed using PhyloBayes MPI v.1.5a [72]. Two parallel runs with four chains were performed, and started from a random topology. The site-heterogeneous CAT-GTR model was used for the analysis, which was originally developed to reduce long-branch attraction artifacts by modelling site-specific features of sequence evolution [73]. Convergence of runs was assessed using bpcomp program implemented in PhyloBayes to ensure that analyses had reached stationarity and that the maxdiff value was less than 0.1. Trees sampled after the burn-in from the two runs were combined and used to build a 50% majority rule consensus tree, with bpcomp program. 

To investigate the potential effect of long-branch taxa on tree reconstruction, we compiled a reduced taxon dataset, namely the dataset of 48taxa_PCGRNA. In which, the long-branched *Rhysodes* sp. was removed. The same phylogenetic analyses were repeated with the dataset of 48taxa_PCGRNA. The sequence alignments supporting the phylogenetic results generated in this article are available in figshare (DOI: 10.6084/m9.figshare.11669280). 

## 3. Results

### 3.1. Next-Generation Sequencing Output and Mitogenome Organization 

In total, 4,110,380 mapped bases were generated by sequencing from the Illumina HiSeq2500. The mean base coverage of the mitochondrial contig was 248-fold. The nearly complete mitogenome of *A. aulica* was 16,646 bp in length. The only gap occurred in the putative control region.

The obtained mitogenome of *A. aulica* consisted of 13 protein-coding genes, 22 tRNA genes, two rRNA genes and a partial control region (Figure 1). There are 23 genes encoded on the heavy strand, while the remaining 14 genes encoded on the light strand. The organization of *A. aulica* mitogenome was compact, because only 29 bp gene overlaps were identified in 11 gene junctions, with the length ranging from one to seven nucleotides. A total of 116 bp intergenic spacers were found in 16 positions, which had the lengths ranging from one to 32 bp. The largest intergenic regions (32 bp) lied between *trnW* and *trnC*. The average nucleotide composition of the full mitogenome sequence was: A = 41.2%, T = 39.2%, C = 11.5% and G = 8.0%, which shows a strong bias towards A and T nucleotides (80.4%). In the *A. aulica* mitogenome, the AT-skew is 0.025, whereas the GC-skew is −0.179 (Table 3).

### 3.2. Protein-Coding Gene

The protein-coding genes had a total length of 11,194 bp, which encoded 3719 amino acid residues and the 37 bp stop codons. Nine out of 13 protein-coding genes were encoded on the heavy strand, while the remaining four were encoded on the light strand. All the protein-coding genes started with the typical ATN codons, except for the *cox1* gene. The start codon ATT was used for *nad3*, *nad5*, *nad4l* and *atp8*, ATG for *cox2*, *cox3*, *atp6*, *nad4* and *cob*, ATA for *nad1*, *nad2* and *nad6*. The *cox1* gene was initiated with the unusual CGA. The *cox2* gene used a single T as the stop codon, while the rest of protein-coding genes ended with the complete termination codon TAA or TAG. The relative synonymous codon usage (RSCU) of *A. aulica* mitogenome are presented in Figure S1 and Table S2. The results showed that UUA (Leu2), AUU (Ile), UUU (Phe), AUA (Met) and AAU (Asn) were the five most frequently used codons. It was obvious that all of them were AT-rich codons. The A+T content of protein-coding genes was 78.5%, and the third codon positions had the highest A + T content (Table 3).

### 3.3. Transfer RNAs

Twenty-two tRNA genes were identified in the mitogenome of *A. aulica* and ranged in length from 64 bp to 72 bp. The full length of tRNA genes was 1478 bp. Fourteen tRNA genes were located on the heavy strand, and the remaining eight were encoded on the light strand. The inferred secondary structures for tRNAs are provided in Figure S2. With the exception of *trnS1*, all tRNA genes can be folded into the typical cloverleaf secondary structure. In the structure of *trnS1*, the dihydrouridine arm was replaced by a simple loop, which is a common character in most of insect mitogenomes published.

### 3.4. Ribosomal RNAs

The large ribosomal gene (*rrnL*) was 1293 bp in length, which was located between the *trnL* (CUN) and *trnV*. The small ribosomal gene (*rrnS*) was 699 bp, which was located between *trnV* and the control region. The inferred secondary structures of both *rrnL* and *rrnS* are shown in Figures S3 and S4. The secondary structure of *rrnL* contained five domains (labeled I, II, IV, V and VI) and 50 helices. The *rrnS* gene was composed of three domains (labeled I, II, III) and 30 helices. 

### 3.5. Phylogenetic Analysis

**Figure 2:**
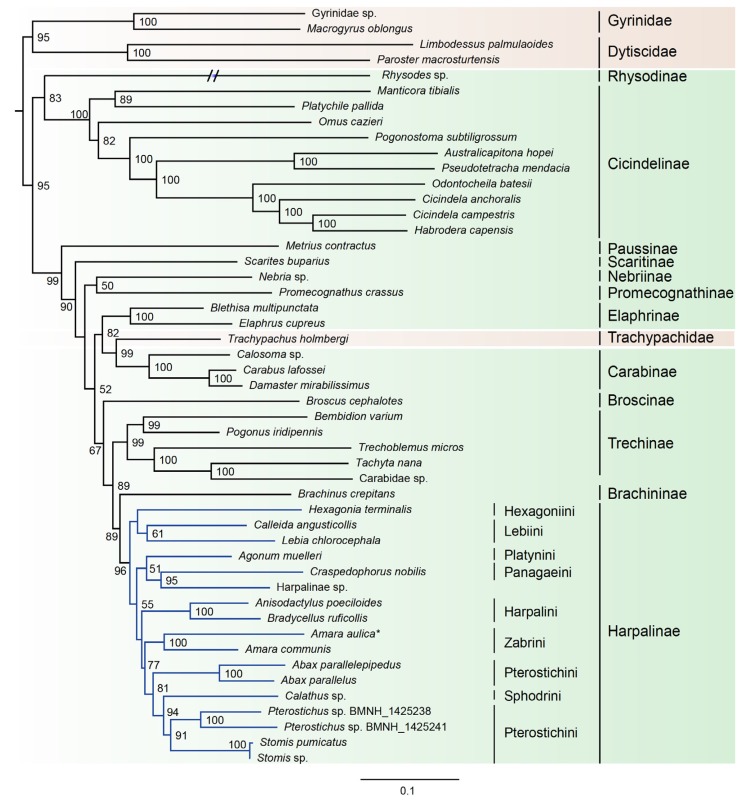
Maximum likelihood tree inferred from the dataset of 49taxa_PCGRNA using IQ-TREE, under the partition schemes and best-fitting models selected by PartitionFinder. Bootstrap support values (≥50) are indicated near the nodes. The branch of *Rhysodes* is depicted as half of its original branch length. Green background indicates the ingroup taxa of Carabidae, and brown indicates the outgroups. Blue lines indicate the Harpalinae. The newly determined *Amara aulica* is emphasized by asterisk.

**Figure 3:**
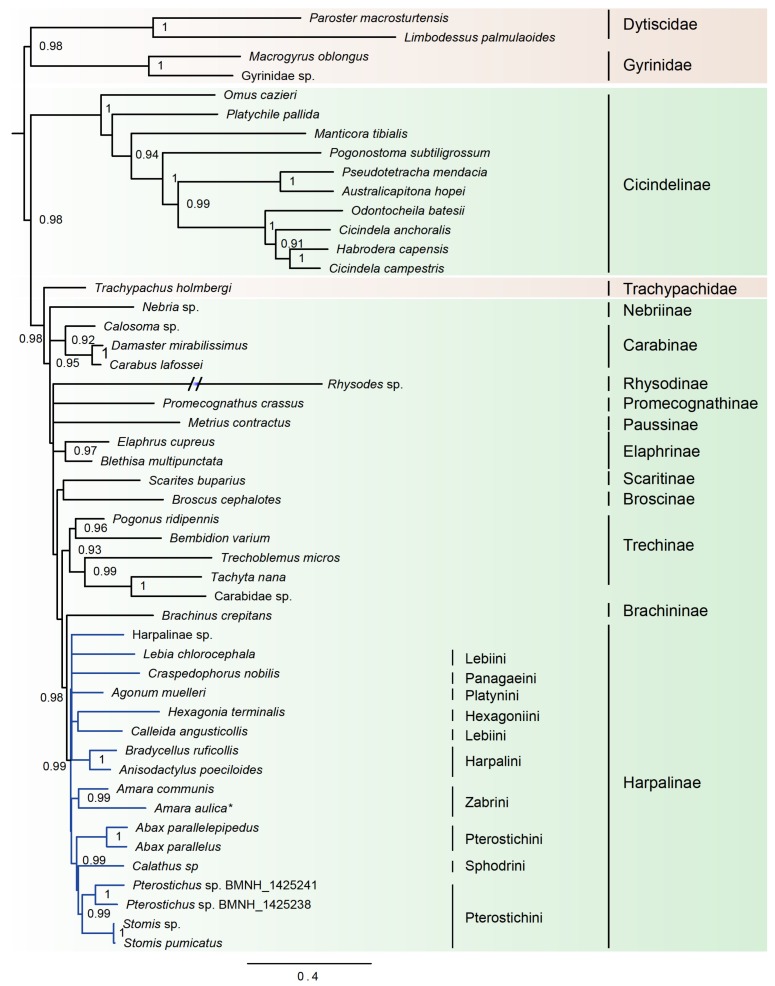
Bayesian tree inferred from the dataset of 49taxa_PCGRNA using PhyloBayes under the site-heterogeneous CAT-GTR model. Poster probability values (≥50) are indicated near the nodes. The branch of *Rhysodes* is depicted as half of its original branch length. Green background indicates the ingroup taxa of Carabidae, and brown indicates the outgroups. Blue lines indicate the Harpalinae. The newly determined *Amara aulica* is emphasized by asterisk.

Based on the results from PartitonFinder, six partition schemes were selected for the dataset of 49taxa_PCGRNA, and the GTR+I+G or GTR+G model was the preferred model for the corresponding partition (Table S1). Both Bayesian trees and ML trees revealed an extremely long terminal branch corresponding to the *Rhysodes* (Figure 2 and Figure 3). Moreover, the placement of *Rhysodes* varied between analyses. In the ML tree under the site-homogeneous GTR model, the *Rhysodes* was retrieved as sister group to Cicindelinae, and both together were sister to the remaining carabid beetle lineages (including Trachypachidae). This branching pattern may be due to long-branch attraction effect. The substitution rate analyses indicated that the *Rhysodes* has been engaged in a process of accelerated rate of evolution, with the highest *ka*/*ks* values among the species analyzed (Table 4). In the long-branch extraction analyses, the removal of the *Rhysodes* did not change the tree topology greatly (Figures S5 and S6). 

The family Trachypachidae always embedded within Carabidae, rendering the latter to be a non-monophyletic assemblage. In the ML analysis based on the dataset of 49taxa_PCGRNA, the Trachypachidae was the sister to the subfamily Carabinae, whereas in the Bayesian analysis based on the same dataset, the Trachypachidae was placed in an intermediate position between the subfamily Cicindelinae and the remaining carabid beetles. 

Within the family Carabidae, four subfamilies with multiple taxon sampling (Cicindelinae, Carabinae, Elaphrinae and Harpalinae) were consistently recovered as monophyletic groups with high support (BP ≥ 96, PP ≥ 0.92). The Cicindelinae were placed as sister group to the remaining ingroup taxa. The monophyly of Trechinae remained elusive due to the ambiguous classification of the exemplar of Carabidae sp. (GenBank accession number: KT696200). A sister group relationship between Brachininae and Harpalinae was strongly supported (BP = 89, PP = 0.98). The phylogenetic positions of the remaining carabid subfamilies were unstable across phylogenetic analyses.

The subfamily Harpalinae had the largest taxon coverage in this study, which allowed us to address some lower taxonomic relationships within this group. The newly sequenced *A. aulica* was strongly supported as a sister to *Amara communis* (BP = 100, PP = 0.99). At the tribe level, the Pterostichini was found to be paraphyletic, with Sphodrini embedded therein. The Zabrini formed a sister group to the clade comprising Pterostichini and Sphodrini. The relationships between the rest of harpaline tribes remained largely unresolved in the Bayesian trees (Figure 3, Figure S6). In contrast, ML trees elucidated a clearer relationship: the intermediate position of Harpalini, and a sister-group relationship of Hexagoniini with Lebiini (Figure 2, Figure S5).

## 4. Discussion

Previous studies have shown that the site-heterogeneous CAT-GTR model implemented in Bayesian analysis can effectively suppress the long-branch attraction artefacts in the animal phylogeny [52,74,75,76,77]. The long-branched Rhysodinae was pulled toward a more derived position and away from the Cicindelinae in the PhyloBayes trees. However, analyses using the site-heterogeneous CAT-GTR model showed limited resolution on the subfamily relationships among Promecognathinae, Paussinae and Elaphrinae (Figure 3, Figure S6). 

The family Carabidae was recovered as non-monophyletic, with respect to Trachypachidae (Figure 2 and Figure 3, Figures S5 and S6). Maddison et al. (2009) [24] supported the nested placement of Trachypachidae within a monophyletic Geadephaga, based on the nuclear gene sequences. However, the sister group of Trachypachidae within Geadephaga is undetermined. Trachypachids were placed with Carabitae, migadopines, elaphrines or a large clade comprising the majority of carabids [24]. In the study of Mckenna et al. (2015) [42] with expanding nuclear gene markers, the placement of Trachypachidae was still unclear. It clustered with *Calosoma* (Carabidae) or other Carabini [42]. These branching patterns resulted in a paraphyletic Carabidae. The similar situation was revealed in the current analyses based on the mitogenome sequence data. 

In the Bayesian tree from 49taxa_PCGRNA, the Cicindelinae was placed as sister to all other carabids (including Trachypachidae). This reconstruction was consistent with some previous studies [23,35,36,41], but contradicted the more derived position recognized by the studies of Beutel et al. (2006) [17] and Hunt et al. (2007) [33]. The “CRPS quartet” (Cicindelidae + Rhysodinae + Paussinae + Scaritinae) inferred in the previous studies [23,24,38,40] was never recovered in the present study.

Within Carabidae, the subfamily relationships changed depending on analyses. Compared with ML trees, the deep divergences among several carabid subfamilies were unresolved in the Bayesian trees (Figure 3, Figure S6). Tree topology comprising very short internodes of early divergences occurred frequently in phylogenetic analysis [78,79]. Lack of resolution may be owing to non-optimal substitution rates, insufficient and conflicting phylogenetic signal. The short internal branches associated with the deep-level relationships of carabids (the large polytomy) also emerged in the prior studies [23]. The authors attributed this to inappropriate methods of inference. Rogue taxa may be another factor leading to weak nodal support and very short internal branches [38]. In addition, rapid radiation of beetle insects may explain the generally short diverging nodes between major groupings at the base of the carabid tree. A large clade comprising Trechinae, Brachininae and Harpalinae was consistently recovered in all analyses. The Brachininae formed a sister group to Harpalinae, both of which were sister to Trechinae. These two sister group relationships were strongly supported (BP ≥ 89, PP ≥ 0.98). This result was concordant with previous studies [23,45].

## 5. Conclusions

The Harpalinae is a megadiverse group within the family Carabidae. However, mitogenome sequences available for Harpaline are very limited. Here, we presented the detailed description of the nearly complete mitogenome of *A. aulica* (Carabidae, Harpalinae). In this mitogenome, gene order and content are consistent with the hypothesized ancestral insect [49]. The new mitogenome sequence was added to investigate the phylogenetic relationships among carabid beetles. The results supported the Carabidae to be a non-monophyletic group with respect to the Trachypachidae. Four subfamilies within Carabidae were strongly supported, namely Cicindelinae, Carabinae, Elaphrinae and Harpalinae. The Cicindelinae was retrieved as sister to all other carabid lineages. The Trechinae (including Carabidae sp.-KT696200) formed a sister group to the clade of (Brachininae + Harpalinae). These results demonstrated that mitogenome sequences can be useful for resolving the subfamily relationships of Carabidae.

## Figures and Tables

**Table 1:** Organization of the *Amara aulica* mitochondrial genome.

Gene	Strand	Location	Length (bp)	Anti Codon	Start Codon	Stop Codon	OVL/ITS
trnI(gat)	H	1-65	65	GAU	-	-	3
trnQ(ttg)	L	69-137	69	UUG	-	-	-1
trnM(cat)	H	137-205	69	CAU	-	-	0
nad2	H	206-1231	1026	-	ATA	TAA	1
trnW(tca)	H	1233-1300	68	UCA	-	-	32
trnC(gca)	L	1333-1397	65	GCA	-	-	2
trnY(gta)	L	1400-1467	68	GUA	-	-	1
cox1	H	1469-3004	1536	-	CGA	TAA	-5
trnL2(taa)	H	3000-3065	66	UAA	-	-	1
cox2	H	3067-3754	688	-	ATG	T	0
trnK(ctt)	H	3755-3825	71	CUU	-	-	0
trnD(gtc)	H	3826-3892	67	GUC	-	-	0
atp8	H	3893-4054	162	-	ATT	TAA	-7
atp6	H	4048-4725	678	-	ATG	TAA	8
cox3	H	4734-5522	789	-	ATG	TAA	2
trnG(tcc)	H	5525-5590	66	UCC	-	-	0
nad3	H	5591-5944	354	-	ATT	TAA	0
trnA(tgc)	H	5945-6012	68	UGC	-	-	-1
trnR(tcg)	H	6012-6078	67	UCG	-	-	4
trnN(gtt)	H	6083-6146	64	GUU	-	-	0
trnS1(gct)	H	6147-6212	66	GCU	-	-	2
trnE(ttc)	H	6215-6281	67	UUC	-	-	-2
trnF(gaa)	L	6280-6347	68	GAA	-	-	-1
nad5	L	6347-8077	1731	-	ATT	TAA	0
trnH(gtg)	L	8078-8145	68	GUG	-	-	-1
nad4	L	8145-9485	1341	-	ATG	TAA	-7
nad4l	L	9479-9769	291	-	ATT	TAA	2
trnT(tgt)	H	9772-9835	64	UGU	-	-	0
trnP(tgg)	L	9836-9902	67	UGG	-	-	10
nad6	H	9913-10428	516	-	ATA	TAA	-1
cob	H	10428-11567	1140	-	ATG	TAG	-2
trnS2(tga)	H	11566-11634	69	UGA	-	-	16
nad1	L	11651-12592	942		ATA	TAG	10
trnL1(tag)	L	12603-12666	64	UAG	-	-	4
rrnL	L	12671-13963	1293		-	-	18
trnV(tac)	L	13982-14053	72	UGC	-	-	-1
rrnS	L	14053-14751	699		-	-	0
Control region	-	14752-16646	1895	-	-	-	-

Abbreviations: H, the heavy strand; L, the light strand; OVL, overlaps (minus number); ITS, intergenic sequence.

**Table 2:** List of the species included in this study.

Family	Subfamily	Tribe	Species	Accession Number
Carabidae	Brachininae	Brachinini	*Brachinus crepitans*	JX412826
Carabidae	Broscinae	Broscini	*Broscus cephalotes*	MF497819
Carabidae	Carabinae	Carabini	*Calosoma* sp.	GU176340
Carabidae	Carabinae	Carabini	*Carabus lafossei*	NC_036507
Carabidae	Carabinae	Carabini	*Damaster mirabilissimus*	GQ344500
Carabidae	Cicindelinae	Cicindelini	*Cicindela anchoralis*	NC_03819
Carabidae	Cicindelinae	Cicindelini	*Cicindela campestris*	MF497823
Carabidae	Cicindelinae	Cicindelini	*Habrodera capensis*	JX412824
Carabidae	Cicindelinae	Cicindelini	*Odontocheila batesii*	MF497818
Carabidae	Cicindelinae	Collyridini	*Pogonostoma subtiligrossum*	MF497820
Carabidae	Cicindelinae	Manticorini	*Manticora tibialis*	MF497821
Carabidae	Cicindelinae	Megacephalini	*Omus cazieri*	MF497813
Carabidae	Cicindelinae	Megacephalini	*Platychile pallida*	MF497814
Carabidae	Cicindelinae	Megacephalini	*Australicapitona hopei*	MF497816
Carabidae	Cicindelinae	Megacephalini	*Pseudotetracha mendacia*	MF497815
Carabidae	Elaphrinae	Elaphrini	*Blethisa multipunctata*	KX087243
Carabidae	Elaphrinae	Elaphrini	*Elaphrus cupreus*	KX087286
Carabidae	Harpalinae	Harpalini	*Anisodactylus poeciloides*	KX087236
Carabidae	Harpalinae	Harpalini	*Bradycellus ruficollis*	KX087248
Carabidae	Harpalinae	Hexagoniini	*Hexagonia terminalis*	JX412768
Carabidae	Harpalinae	Hexagoniini	*Lebia chlorocephala*	KX087304
Carabidae	Harpalinae	Lebiini	*Calleida angusticollis*	JX412855
Carabidae	Harpalinae	Panagaeini	*Craspedophorus nobilis*	JX412738
Carabidae	Harpalinae	Platynini	*Agonum muelleri*	JX412835
Carabidae	Harpalinae	Pterostichini	*Abax parallelepipedus*	KT876877
Carabidae	Harpalinae	Pterostichini	*Abax parallelus*	KX087231
Carabidae	Harpalinae	Pterostichini	*Pterostichus* sp. BMNH 1425238	KT876910
Carabidae	Harpalinae	Pterostichini	*Pterostichus* sp. BMNH 1425241	KT876909
Carabidae	Harpalinae	Pterostichini	*Stomis pumicatus*	KX087349
Carabidae	Harpalinae	Pterostichini	*Stomis* sp.	KT876914
Carabidae	Harpalinae	Sphodrini	*Calathus* sp.	KT876884
Carabidae	Harpalinae	Zabrini	*Amara aulica*	MN335930
Carabidae	Harpalinae	Zabrini	*Amara communis*	KX035135
Carabidae	Harpalinae	-	Harpalinae sp.	JX412794
Carabidae	Nebriinae	Nebriini	*Nebria* sp.	KT876906
Carabidae	Paussinae	Metriini	*Metrius contractus*	MF497817
Carabidae	Promecognathinae	Promecognathini	*Promecognathus crassus*	JX313665
Carabidae	Rhysodinae	-	*Rhysodes* sp.	KX035156
Carabidae	Scaritinae	Scaritini	*Scarites buparius*	MF497822
Carabidae	Trechinae	Bembidiini	*Bembidion varium*	KX087242
Carabidae	Trechinae	Bembidiini	*Tachyta nana*	KX035142
Carabidae	Trechinae	Pogonini	*Pogonus iridipennis*	KX087338
Carabidae	Trechinae	Trechini	*Trechoblemus micros*	KX035144
Carabidae	-	-	Carabidae sp.	KT696200
Dytiscidae	-	-	*Paroster macrosturtensis*	MG912995
Dytiscidae	-	-	*Limbodessus palmulaoides*	NC_037749
Gyrinidae	-	-	Gyrinidae sp.	JX412840
Gyrinidae	-	-	*Macrogyrus oblongus*	FJ859901
Trachypachidae	-	-	*Trachypachus holmbergi*	EU877954

Note: Bold indicates the species newly sequenced in this study.

**Table 3:** Nucleotide composition of the *Amara aulica* mitochondrial genome.

	T%	C%	A%	G%	A + T%	AT-skew	GC-skew
Whole mitogenome	39.2	11.5	41.2	8	80.4	0.025	−0.179
Protein-coding genes	44.3	10.3	34.2	11.1	78.5	−0.129	0.037
1st codon positions	38	10.2	35.3	16.5	73.3	−0.037	0.236
2nd codon positions	47.9	17.3	21	13.9	68.9	−0.390	−0.109
3rd codon positions	47.1	3.6	46.2	3.1	93.3	−0.010	−0.075
tRNA genes	40.2	7.7	40.7	11.4	80.9	0.006	0.194
rRNA genes	42.6	6.1	39.7	11.7	82.2	−0.035	0.316

**Table 4:** The substitution rate analyses conducted by DnaSP.

Species	*ks*	*ka*	*ka*/*ks*
*Abax parallelepipedus*	0.839	0.101	0.121
*Abax parallelus*	0.788	0.102	0.129
*Agonum muelleri*	0.727	0.077	0.107
*Amara aulica*	0.727	0.098	0.135
*Amara communis*	0.646	0.089	0.138
*Anisodactylus poeciloides*	0.746	0.089	0.120
*Australicapitona hopei*	2.243	0.119	0.053
*Bembidion varium*	0.815	0.101	0.124
*Blethisa multipunctata*	0.769	0.087	0.113
*Brachinus crepitans*	0.790	0.114	0.144
*Bradycellus ruficollis*	0.714	0.086	0.120
*Broscus cephalotes*	0.855	0.156	0.182
*Calathus* sp.	0.787	0.088	0.112
*Calleida angusticollis*	0.738	0.087	0.119
*Calosoma* sp.	0.915	0.086	0.093
Carabidae sp.	0.729	0.102	0.140
*Carabus lafossei*	0.786	0.088	0.112
*Cicindela anchoralis*	1.197	0.117	0.097
*Cicindela campestris*	1.072	0.117	0.109
*Craspedophorus nobilis*	0.823	0.089	0.108
*Damaster mirabilissimus*	0.818	0.091	0.111
*Elaphrus cupreus*	0.780	0.089	0.115
Gyrinidae sp.	0.980	0.116	0.118
*Habrodera capensis*	0.951	0.115	0.121
Harpalinae sp.	0.816	0.094	0.115
*Hexagonia terminalis*	0.713	0.103	0.144
*Lebia chlorocephala*	0.810	0.094	0.116
*Limbodessus palmulaoides*	1.060	0.124	0.117
*Macrogyrus oblongus*	1.004	0.120	0.120
*Manticora tibialis*	1.044	0.141	0.135
*Metrius contractus*	0.931	0.125	0.134
*Nebria* sp.	0.765	0.096	0.126
*Odontocheila batesii*	1.043	0.114	0.109
*Omus cazieri*	0.878	0.107	0.121
*Paroster macrosturtensis*	1.110	0.115	0.103
*Platychile pallida*	0.876	0.117	0.133
*Pogonostoma subtiligrossum*	0.880	0.120	0.137
*Pogonus iridipennis*	0.645	0.092	0.143
*Promecognathus crassus*	0.936	0.117	0.125
*Pseudotetracha mendacia*	1.602	0.116	0.072
*Pterostichus* sp. BMNH_1425238	0.704	0.095	0.135
*Pterostichus* sp. BMNH_1425241	0.731	0.096	0.131
*Rhysodes* sp.	1.013	0.208	0.205
*Scarites buparius*	0.817	0.109	0.133
*Stomis pumicatus*	0.662	0.097	0.146
*Stomis* sp.	0.662	0.096	0.144
*Tachyta nana*	0.715	0.100	0.139
*Trachypachus holmbergi*	0.719	0.102	0.142
*Trechoblemus micros*	0.724	0.117	0.162

## Supplementary Materials

The following are available online at https://www.mdpi.com/2073-4425/11/2/181/s1, Figure S1: Relative synonymous codon usage (RSCU) in the *Amara aulica* mitochondrial genome, Figure S2: Putative secondary structures of the 22 tRNA genes from *Amara aulica*, Figure S3: Putative rrnL secondary structure in the Amara aulica mitochondrial genome, Figure S4: Putative rrnS secondary structure in the Amara aulica mitochondrial genome, Figure S5: Maximum likelihood tree inferred from the dataset of 48taxa_PCGRNA using IQ-TREE under the best-fitting models, Figure S6: Bayesian tree inferred from the dataset of 48taxa_PCGRNA using PhyloBayes under the site-heterogeneous CAT-GTR model, Table S1: The best partitioning schemes selected by PartitionFinader for the dataset of (A) 49taxa_PCGRNA and (B) 48taxa_PCGRNA, Table S2: Codon usage of protein-coding genes in the *Amara aulica* mitochondrial genome.
